# Optimization of Black Boar Sperm Cryopreservation Efficiency with Antioxidant-Rich Plant Extracts from Djulis (*Chenopodium formosanum*)

**DOI:** 10.3390/ani15121798

**Published:** 2025-06-18

**Authors:** Wenchi Hsu, Tzuche Lin, Shenchang Chang, Minjung Lin, Chaowei Huang, Perngchih Shen, Chihjen Chou, Shaoyu Peng

**Affiliations:** 1 Department of Animal Science, National Pingtung University of Science and Technology, Pingtung 912301, Taiwan; m11126004@office365.npust.edu.tw (W.H.); pcshen@mail.npust.edu.tw (P.S.); 2 Department of Plant Industry, National Pingtung University of Science and Technology, Pingtung 912301, Taiwan; tclin@mail.npust.edu.tw; 3 Southern Region Branch, Taiwan Livestock Research Institute, Ministry of Agriculture, Executive Yuan, Pingtung 912301, Taiwan; macawh@mail.tlri.gov.tw; 4 Bachelor of Scientific Agriculture, National Pingtung University of Science and Technology, Pingtung 912301, Taiwan; mjlin63@mail.npust.edu.tw; 5 Department of Tropical Agriculture and International Cooperation, National Pingtung University of Science and Technology, Pingtung 912301, Taiwan; cwhuang@g4e.npust.edu.tw

**Keywords:** antioxidant, boar semen, cryopreservation, djulis (*Chenopodium formosanum*)

## Abstract

The cryopreservation of boar sperm effectively extends its storage period but often leads to increased reactive oxygen species (ROS) production, compromising sperm quality. Plant extracts, rich in bioactive compounds such as polyphenols and flavonoids, have been shown to reduce ROS. Djulis (*Chenopodium formosanum*), also known as the “ruby of cereals”, is nutritionally rich and holds potential as a cryoprotective additive. This study aimed to determine the optimal concentration of extracts from different parts of djulis, including unhulled seeds and stems, for effective boar semen cryopreservation. Fresh semen from Taiwan indigenous boars was diluted with a modified GLT-cryoprotectant extender containing glycerol, low-density lipoprotein (LDL), and trehalose. The experimental groups included DSS25, DSS50, DS25, and DS50—representing djulis unshelled seed at 25 mg/mL and 50 mg/mL, and djulis stem at 25 mg/mL and 50 mg/mL in distilled water, respectively—alongside a control group without additives. Post-thaw assessments included sperm motility, kinetic parameters, viability, acrosome integrity, and the antioxidant properties of djulis extracts, such as DPPH radical scavenging activity and total phenolic acid content. Results showed that total motility (TM) was significantly higher in the DSS25 (48.8 ± 3.9), DSS50 (49.0 ± 6.7), and DS50 (49.0 ± 2.4) groups compared to the control group (31.3 ± 4.8). Similarly, progressive motility (PM) was significantly improved in DSS25 (27.5 ± 2.7) and DSS50 (26.8 ± 4.1) versus the control (12.8 ± 3.2). However, for straightness (STR), the control group (87.8 ± 1.3) exhibited significantly higher values than the DS50 group (83.5 ± 1.3) (*p* < 0.05). Viability and acrosome integrity showed no significant differences across groups. In conclusion, djulis extracts positively influence sperm motility and forward movement, with 1% djulis extract confirmed to enhance the quality of cryopreserved semen. Future research will focus on determining the optimal dosage of djulis extract for improved cryopreservation outcomes.

## Simple Summary

This study explored the use of djulis (*Chenopodium formosanum*) extracts as a potential additive to improve boar sperm cryopreservation. The cryopreservation process can increase reactive oxygen species (ROS), negatively affecting sperm quality. The research aimed to identify the optimal concentration of djulis extracts from unhulled seeds and stems for enhancing sperm quality during cryopreservation. Fresh semen from Taiwan indigenous boars was diluted with a modified cryoprotectant extender and supplemented with djulis extracts at different concentrations. The study assessed sperm motility, viability, acrosome integrity, and antioxidant properties post-thaw. Results showed that djulis extracts, particularly from unhulled seeds, improved sperm motility, with significant enhancements in total and progressive motility. The findings suggest that 1% djulis extract improves sperm quality, and further studies will focus on refining the optimal dosage for better cryopreservation results.

## 1. Introduction

The cryopreservation of boar sperm is an efficient strategy for the long-term preservation of valuable genomic material [1]. However, this process can negatively impact sperm due to cold shock, osmotic stress, and oxidative stress (OS) caused by reactive oxygen species (ROS) and reactive nitrogen species (RNS), which reduce the lipid content of sperm membranes [2,3,4]. Boar sperm is particularly sensitive to ROS due to the high concentration of polyunsaturated fatty acids (PUFAs) in its membranes [3,5].

ROS and RNS exert both positive and negative effects on male fertility. Reactive species such as superoxide anion (⋅O_2_^−^), hydrogen peroxide (H_2_O_2_), nitric oxide (NO), and peroxynitrite (-ONOO^−^) significantly influence sperm motility, viability, and physiology by interacting with membrane lipids, proteins, and DNA [6,7,8]. At low concentrations, these molecules play crucial roles in sperm capacitation, hyperactivation, the acrosome reaction, and sperm–oocyte fusion. However, excessive ROS production induces OS, leading to membrane damage, lipid peroxidation (LPO), DNA oxidation, and apoptosis, ultimately resulting in infertility [9,10,11].

Seminal plasma contains numerous antioxidant enzymes, including superoxide dismutase (SOD) and glutathione peroxidase (GPx), which exhibit the highest activity in neutralizing ROS. However, semen cryopreservation protocols often involve centrifugation to remove seminal plasma, thereby eliminating these protective enzymes and exacerbating the harmful effects of free radicals [12,13].

Recently, plant extracts have gained attention as natural additives for protecting and enhancing sperm function during storage. Many plants are rich in antioxidants that scavenge ROS and mitigate OS-related damage [14]. Among them, djulis (*Chenopodium formosanum*), a herbaceous plant in the Amaranthaceae family, contains antioxidants such as phenolic compounds and betacyanins, with rutin being the predominant polyphenol [15,16]. Djulis has demonstrated various biological activities, including hepatoprotection [17,18], anti-adipogenesis [19], and the regulation of hyperlipidemia and hyperglycemia [20].

This study aims to investigate the antioxidant properties of djulis and evaluate its effects on the quality of boar sperm after cryopreservation.

## 2. Materials and Methods

### 2.1. Preparation of Djulis Extracts

**Figure 1:**
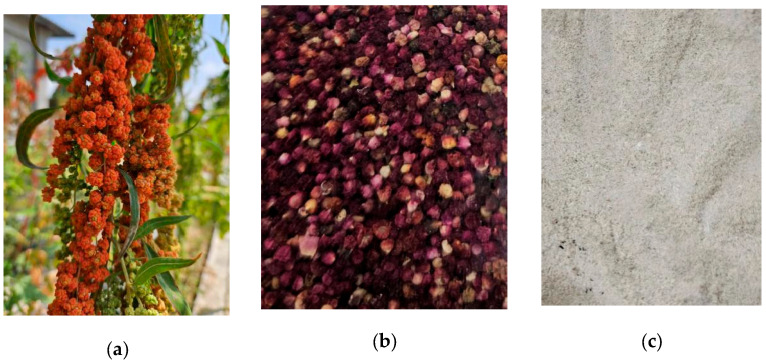
Morphological characteristics of djulis (*Chenopodium formosanum*). (**a**) The whole plant, (**b**) unshelled seeds, (**c**) stem powder.

Djulis unshelled seeds and stems were obtained from the National Pingtung University of Science and Technology (NPUST) (Figure 1). The seeds and stems were ground into powder using a grinder (RT-02A, Rong Cong Precision Technology Co., Ltd., Taichung City, Taiwan), and the resulting powder was passed through a 0.25 mm sieve. To ensure consistency across all experimental treatments, a single large batch of each extract concentration (2.5 g and 5 g of powder per 100 mL of distilled water) was prepared simultaneously. Specifically, these concentrations were achieved by separately mixing 2.5 g and 5 g of the powder with 100 mL of distilled water preheated to 100 °C and allowed to extract for 10 min. After cooling to below 25 °C, the mixture was filtered through filter paper to remove solid residues. The resulting djulis water extracts were then aliquoted and immediately stored at 4 °C until use in the semen cryopreservation experiments. This single-batch preparation aimed to minimize potential variations in extract composition and activity between different experimental repetitions.

### 2.2. Antioxidant Capacity Analysis: High-Performance Liquid Chromatography (HPLC) Assessment

The total phenolic content and 2,2-diphenyl-1-picrylhydrazyl (DPPH) radical scavenging activity of djulis extracts were evaluated using high-performance liquid chromatography (HPLC). The analysis was performed with a Hitachi U-2900 series HPLC system (Tokyo, Japan) equipped with a double-beam spectrophotometer. All samples were tested in triplicate.

### 2.3. Animal and Semen Processing

All black boar (KHAPS Black Pig) semen samples were obtained from the Southern Region Branch of the Taiwan Livestock Research Institute, Ministry of Agriculture, Taiwan. Semen was collected from four healthy and sexually mature boars (8–24 months old) using the gloved-hand method. One ejaculate was collected from each boar. Only the sperm-rich portion was collected into a separate container, and the opening of the container was covered with gauze to filter out the gelatinous part of the semen. The collected semen was then transported to the National Pingtung University of Science and Technology in insulated bags to maintain the temperature and ensure delivery within one hour.

### 2.4. Semen Freezing Process

Experimental extenders. The extender used for semen collection was Beltsville Thawing Solution (BTS), which contained 79.9 g of glucose, 12.7 g of sodium citrate, 2.65 g of EDTA, 2.65 g of sodium bicarbonate, 1.59 g of potassium chloride, and 1000 mL of distilled water [21]. The glycerol-low-density lipoprotein (LDL)-trehalose (GLT) base extender consisted of 9.93% (*v*/*v*) LDL, 89.34% (*v*/*v*) trehalose solution (300 mM), and 0.74% Equex STM, designated as Formulation 1 (F1). Formulation 2 (F2) was identical to F1 but with the addition of 6% glycerol. Semen was diluted with Beltsville Thawing Solution (BTS) at 37 °C and assessed for volume, concentration, and motility using the portable iSperm^®^ Swine (mCASA) system (Aidmics Biotechnology Co. LTD., Taipei, Taiwan). This device transforms an iPad Mini into a handheld microscope, utilizing dedicated software for objective semen analysis [22]. The iSperm system allows for rapid and on-site assessment of various sperm kinetic parameters, including total motility, progressive motility, and concentration [23,24]. Only samples with ≥80% motility and normal morphology were cryopreserved. Boar semen was cryopreserved following a modified protocol adapted from Carvajal et al. (2004) [25]. Semen was cooled to 17 °C over 2 h, centrifuged at 800× *g* for 15 min, and the pellet was re-suspended in Formulation 1 (F1). After further cooling to 4 °C in 2 h, samples were divided into five groups: a control group and four experimental groups supplemented with 1% Formulation 2 (F2) containing djulis water extract (DSS25, DSS50, DS25, DS50). The final sperm concentration was adjusted to 20 × 10^9^ spermatozoa/mL. Spermatozoa were loaded into 0.5 mL straws, sealed, exposed to nitrogen vapor (3 cm above liquid nitrogen) for 15 min, and then immersed in −196 °C liquid nitrogen for storage. Samples were frozen for 7 days before evaluation.

### 2.5. Semen Thawing Process and Quality Assessment

Frozen semen samples were rapidly thawed in a water bath at 50 °C for 12 s. Immediately after thawing, the semen was diluted at a 1:4 ratio (v:v) with a pre-warmed (37 °C) BTS extender. The diluted semen was then maintained at 37 °C for 10 min before subsequent quality assessments. Post-thaw sperm quality, including total motility (TM%), progressive motility (PM%), and various kinetic parameters, was objectively evaluated using the iSperm^®^ Swine system (Aidmics Biotechnology Co. LTD., Taipei, Taiwan). The kinetic parameters assessed included curvilinear velocity (VCL, µm/s), straight-line velocity (VSL, µm/s), average pathway velocity (VAP, µm/s), straightness (STR, %), and linearity (LIN, %).

### 2.6. Evaluation of Sperm Viability

Sperm viability was assessed using a dual fluorescent staining method with SYBR Green I (Sigma, S9430, St. Louis, MO, USA) and propidium iodide (PI; Sigma, P4170, St. Louis, MO, USA). The SYBR Green I working solution (10,000-fold dilution) and PI working solution (2 mg/mL) were prepared in 1× phosphate-buffered saline without Ca^2+^ and Mg^2+^ (mPBS), and stored at −20 °C in the dark until use. Then, 10 µL of semen was mixed with 10 µL of SYBR Green I and 5 µL of PI, then incubated at 37 °C for 1 min. A minimum of 200 sperm were examined under a fluorescence microscope (Olympus BX51, Tokyo, Japan) at 400× magnification. Sperm stained with SYBR Green I/PI were classified as viable or non-viable. Live sperm with intact plasma membranes exhibited green fluorescence in their nuclei, while dead or membrane-compromised sperm displayed red fluorescence. The percentage of viable and non-viable sperm was recorded.

### 2.7. Evaluation of Acrosome Integrity

Sperm acrosome integrity was assessed using fluorescein isothiocyanate-labeled peanut agglutinin (FITC-PNA; Sigma, L7381, St. Louis, MO, USA) staining in conjunction with propidium iodide (PI; Sigma, P4170, St. Louis, MO, USA) to identify viable cells. The FITC-PNA working solution (100 µg/mL) was freshly prepared from a 1 mg/mL stock (stored at −20 °C in the dark) by diluting in 1× phosphate-buffered saline (PBS). The PI working solution was prepared as described in Section 2.6. Thawed semen (50 µL) was washed twice with 450 µL of 1× PBS by centrifugation (600× *g* for 5 min each). The washed sperm pellet was then fixed in 50 µL of a solution containing 4% (*w*/*v*) paraformaldehyde and 1% (*v*/*v*) glutaraldehyde at 4 °C for 12–24 h. After fixation, samples were washed twice with 400 µL PBS by centrifugation (600× *g* for 5 min each) to remove the fixative. The final sperm pellet was resuspended in a staining solution containing 50 µL of PBS and 50 µL of the FITC-PNA working solution. Following incubation in the dark at 37 °C for 10 min, samples were washed twice with PBS (600× *g* for 5 min each) to remove the unbound stain. For the final assessment, the pellet was mixed with 50 µL of PBS and 5 µL of the PI working solution. A 10 µL aliquot was placed on a slide for immediate observation under a fluorescence microscope (Olympus BX51, Tokyo, Japan) at 400× magnification, using a wavelength of 498 nm. A minimum of 200 spermatozoa were randomly assessed per sample. Spermatozoa were classified based on fluorescence: sperm with green fluorescence in the acrosomal region were classified as damaged, while fully red-fluorescent sperm were considered intact. Acrosome integrity (%) was calculated as (intact sperm/total sperm) × 100.

### 2.8. Statistical Assessment

Statistical analysis was performed using SAS 9.4 (SAS Institute, Inc., Cary, NC, USA). Data were expressed as mean ± standard error of the mean (SEM). Prior to primary analysis, the normality of data distribution for each seminal parameter (e.g., sperm motility, viability, acrosome integrity, kinematic parameters) was assessed using the Shapiro–Wilk test. For data that fulfilled the assumptions of normality and homogeneity of variances, one-way analysis of variance (ANOVA) was employed to compare means among different treatment groups. Following a significant ANOVA result (*p* < 0.05), Duncan’s multiple range test was used for post-hoc pairwise comparisons. Given our experimental design involving four biological replicates (individual boars) and four technical replicates for each post-thaw semen sample, the four technical replicates were averaged to obtain a single value per boar per treatment group. These averaged values from the four boars were then subjected to the aforementioned statistical analyses to ensure that statistical inferences were based on biological replicates. Statistical significance was set at *p* < 0.05.

## 3. Results

### 3.1. Quantification of Antioxidant Compounds in Djulis Extracts

**Figure 2:**
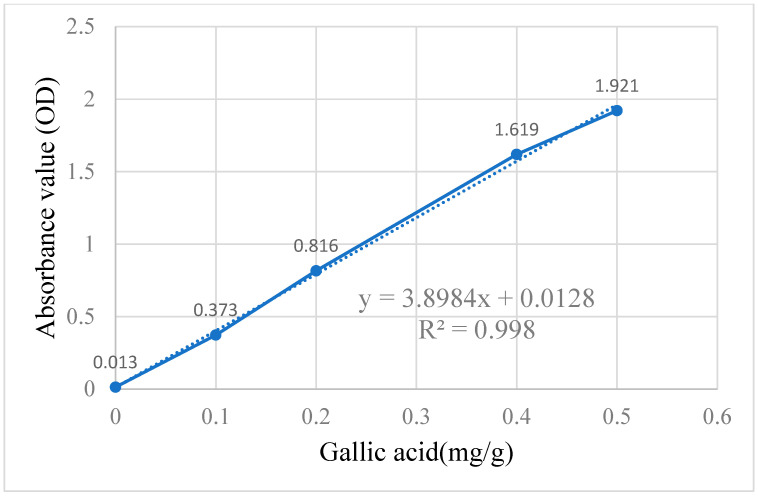
A calibration line was established using gallic acid for the determination of total polyphenols. *X*-axis represents GAE (mg/g) and the *Y*-axis represents the absorbance value (OD). The solid line connects the experimental data points, and the dotted line represents the linear regression (best-fit line) used for calibration.

Total polyphenol content, a key indicator of antioxidant capacity, was measured using gallic acid as a reference for calibration (Figure 2). The X-axis represents gallic acid equivalents (GAE) (mg/g), while the Y-axis indicates absorbance (optical density, OD). Based on this curve, the total polyphenol content in djulis extracts was calculated. As shown in Table 1, the polyphenol content (GAE mg/g) of DSS25, DSS50, DS25, and DS50 was 0.155 ± 0.008, 0.270 ± 0.007, 0.061 ± 0.005, and 0.106 ± 0.015, respectively. DSS50 exhibited the highest polyphenol content, followed by DSS25, DS50, and DS25 in descending order.

### 3.2. Quantification of DPPH Radical Scavenging Activity in Djulis Extracts

The DPPH assay was used to assess the free radical scavenging activity of djulis extracts. As shown in Table 2, all four extracts demonstrated scavenging ability, with efficiencies of 36.8 ± 23.8%, 56.8 ± 19.1%, 52.9 ± 3.1%, and 32.0 ± 2.3% for DSS25, DSS50, DS25, and DS50, respectively. DSS50 and DS50 exhibited higher scavenging efficiencies (50–60%), whereas DSS25 and DS25 ranged between 30–40%, with no significant differences among groups. These results highlight the antioxidant potential of djulis extracts.

### 3.3. Effects of Djulis Extract Supplementation on the Motility and Kinetic Parameters of Freeze–Thawed Black Pig Spermatozoa

This study evaluated the impact of djulis extract supplementation in the freezing medium on post-thaw sperm motility and kinetic parameters (Table 3). Results showed that total motility (TM) was significantly higher in the DSS25 (48.8 ± 3.9), DSS50 (49.0 ± 6.7), and DS50 (49.0 ± 2.4) groups compared to the control group (31.3 ± 4.8). Similarly, progressive motility (PM) was significantly improved in DSS25 (27.5 ± 2.7) and DSS50 (26.8 ± 4.1) versus the control (12.8 ± 3.2). However, for straightness (STR), the control group (87.8 ± 1.3) exhibited significantly higher values than the DS50 group (83.5 ± 1.3) (*p* < 0.05).

### 3.4. Effects of Djulis Extracts Supplementation in the Freezing Medium on the Viability and Acrosome Integrity of Freeze–Thawed Black Pig Spermatozoa

This study examined the impact of djulis extract supplementation on post-thaw sperm viability and acrosome integrity (Table 4). Results revealed no significant differences in either viability or acrosome integrity between the control and treatment groups.

## 4. Discussion

This study investigated the potential of djulis (*Chenopodium formosanum*) aqueous extracts as a natural antioxidant supplement in the cryopreservation extender for black boar semen. Our primary finding demonstrates that djulis extracts significantly improve post-thaw sperm motility, particularly total and progressive motility, at specific concentrations. This discussion will delve into the underlying mechanisms, contextualize our findings within the existing literature, highlight the theoretical and practical implications of using natural antioxidants in semen cryopreservation, and acknowledge the limitations of the current study.

Sperm cryopreservation inevitably induces oxidative stress due to increased reactive oxygen species (ROS) production and reduced antioxidant defense, leading to lipid peroxidation, DNA damage, and decreased post-thaw sperm quality [11,26]. Plant extracts have emerged as promising alternatives to conventional, often costly, synthetic antioxidants for sperm preservation, such as epicatechin gallate, quercetin, and aspartic acid [14,27,28,29]. The economic feasibility of isolated compounds is often a limiting factor for large-scale application in semen extenders. Moreover, unlike individual compounds, whole plant extracts offer a synergistic blend of diverse secondary metabolites (e.g., phenols, flavonoids, terpenes) that possess broad-spectrum antioxidant and antimicrobial properties, potentially offering more comprehensive protection against cryoinjuries [14,30,31].

Djulis, an indigenous Taiwanese grain, has been recognized for its various health-promoting properties, including hepatoprotective, anti-lipogenic, and lipid- and glucose-regulating effects [15,16,19,20]. However, its specific impact on reproductive cells, particularly sperm cryopreservation, has remained largely unexplored prior to this study. Our preliminary antioxidant analysis confirmed that the type and concentration of djulis extract significantly influenced its antioxidant capacity and total polyphenol content [32]. The observed strong antioxidant activity and high polyphenol content at specific concentrations (e.g., 25 mg/mL and 50 mg/mL) in djulis extracts underscore their intrinsic potential for mitigating oxidative stress during semen cryopreservation [33,34]. These natural bioactive compounds likely contribute to enhanced semen quality post-thaw by protecting the plasma membrane against ROS and activating the endogenous antioxidant enzyme system [1,4,9,11,12]. This positions djulis as a promising natural additive for future research in reproductive cryobiology [1].

Our study demonstrated that supplementing the cryopreservation extender with djulis extracts significantly improved post-thaw sperm motility in black boars. Specifically, the concentrations of 25 mg/mL and 50 mg/mL of djulis seed extract (DSS) significantly enhanced both total and progressive motility. While 50 mg/mL of djulis stem extract (DS) also showed an improvement in progressive motility, its effect was less pronounced compared to the seed extracts, suggesting a dose- and source-dependent efficacy that merits further investigation. These findings suggest that djulis extracts, particularly from seeds, can effectively mitigate cryoinjuries and enhance sperm quality after cryopreservation. The optimal concentrations (25 mg/mL and 50 mg/mL DSS) highlight their potential for improving cryopreservation efficiency in boar semen.

Regarding the observed trends where the control group exhibited numerically higher viability and acrosome integrity compared to treatment groups, despite having lower motility, several hypotheses can be considered. Firstly, it is possible that while djulis extracts are highly effective in preserving sperm motility by protecting the flagellar apparatus and energy metabolism pathways, their protective effects on the plasma membrane integrity and acrosome might be less direct or require different concentrations or specific compounds. Motility is a highly sensitive parameter to oxidative stress and cryoinjury, and thus may show more immediate and pronounced improvements with antioxidant supplementation. Secondly, it is plausible that different mechanisms are at play: while antioxidants can reduce oxidative damage, high concentrations of plant extracts might introduce other factors (e.g., osmotic effects, direct interactions with membrane components) that subtly influence viability or acrosome integrity without reaching statistical significance. Future studies could explore a broader range of concentrations, different extraction methods, or specific isolated compounds to identify agents that more comprehensively protect all sperm parameters.

These results are consistent with a growing body of literature reporting the beneficial effects of various plant-derived antioxidants on sperm cryopreservation across different species, particularly in boars. For instance, studies have shown that adding green tea extract to boar semen cryopreservation media increased sperm viability and membrane function while reducing lipid peroxidation. Similarly, when lemon balm extract was incorporated into boar semen extender, it significantly improved the linearity (LIN) and straightness (STR) of post-thaw sperm motility, with optimal concentrations found to be within 2.5–5 g/L [1,2]. Other studies have demonstrated the positive impact of substances like Rhodiola rosea polysaccharides from Rhodiola rosea [35] or curcumin from turmeric [36] on boar sperm cryopreservation, often reporting improvements in motility and membrane integrity by enhancing antioxidant defenses and reducing lipid peroxidation. Similar to our findings, these studies often attribute improvements in motility to the antioxidant and membrane-stabilizing properties of phenolic compounds and flavonoids [17,18,19]. However, our study distinguishes itself by specifically exploring djulis extracts, a previously underutilized natural resource in this context.

The findings of this study carry significant theoretical and practical implications for the field of animal reproduction. Theoretically, our research adds to the understanding of how natural plant-derived compounds, specifically those from djulis, can combat oxidative stress and improve cellular integrity during the extreme conditions of cryopreservation. It suggests that djulis extracts might offer a novel pathway for protecting sensitive reproductive cells from freeze–thaw damage, expanding the repertoire of natural cryoprotective agents. From a practical perspective, the successful application of djulis extracts could provide an economically viable and readily available alternative to expensive synthetic antioxidants in boar semen extenders. This is particularly relevant for the preservation of genetically valuable breeds like the KHAPS black pig, which accounts for a significant portion of Taiwan’s pig farming industry due to its high-quality meat and maternal reproductive performance [37]. Efficient cryopreservation strategies for this breed are crucial for safeguarding its genetic resources and supporting sustainable breeding programs [38].

Despite these promising findings, our study has certain limitations that warrant consideration and suggest avenues for future research. Firstly, the use of four biological replicates (individual boars), while providing robust technical data, might limit the broader generalizability of our findings due to individual variations in semen quality and response to cryopreservation. Future studies with a larger population of boars would enhance the statistical power and external validity of the results. Secondly, our study focused on in vitro post-thaw sperm quality parameters. Further in vivo studies, such as artificial insemination trials, are essential to validate the actual fertility outcomes of semen cryopreserved with djulis extracts. Additionally, while we confirmed the antioxidant capacity and total polyphenol content, the specific bioactive compounds responsible for the cryoprotective effects within djulis extracts warrant further isolation and characterization. Future research should also explore a wider range of extract concentrations and investigate the long-term storage stability of semen treated with djulis.

## 5. Conclusions

This study underscores the promising role of djulis extracts as a natural additive for enhancing black boar semen cryopreservation efficiency, particularly by improving sperm motility. Future research should investigate the mechanisms underlying these protective effects, explore synergistic combinations with other antioxidants, and assess long-term fertility outcomes. Expanding these applications to other livestock species could further validate their practical use in reproductive biotechnology and breeding programs.

## Figures and Tables

**Table 1:** Total phenolic compound content of djulis extract.

Treatment	DSS25	DSS50	DS25	DS50
Total phenolic content (mg of GAE/g)	0.155 ± 0.008 ^b^	0.270 ± 0.007 ^a^	0.061 ± 0.005 ^d^	0.106 ± 0.015 ^c^

Content of total phenolic (mean ± SEM). *n* = 3. ^a–d^ represents the significant difference (*p* < 0.05). Treament: DSS25 (2.5 mg/mL djulis unshelled seed), DSS50 (5 mg/mL djulis unshelled seed), DS25 (2.5 mg/mL djulis stem), DS50 (5 mg/mL djulis stem).

**Table 2:** Scavenging ability of djulis extract toward DPPH radicals.

Treatment	DSS25	DSS50	DS25	DS50
DPPH radical scavenging ability (%)	36.8 ± 23.8	56.8 ± 19.1	52.9 ± 3.1	32.0 ± 2.3

Percentage of DPPH radical scavenging ability (mean ± SEM). *n* = 3. Treament: DSS25 (2.5 mg/mL djulis unshelled seed), DSS50 (5 mg/mL djulis unshelled seed), DS25 (2.5 mg/mL djulis stem), DS50 (5 mg/mL djulis stem).

**Table 3:** Effect of different concentrations of djulis extract on motility and kinetic parameters of boar thawed semen.

Treatment	TM (%)	PM (%)	VCL (µm/s)	VAP (µm/s)	VSL (µm/s)	STR (%)	LIN (%)
Control	31.3 ± 4.8 ^b^	12.8 ± 3.2 ^b^	53.3 ± 3.1	41.5 ± 2.3	37.3 ± 2.2	87.8 ± 1.3 ^a^	69.5 ± 1.9
DSS25	48.8 ± 3.9 ^a^	27.5 ± 2.7 ^a^	61.8 ± 2.3	48.3 ± 1.8	42.5 ± 1.3	86.8 ± 0.8 ^ab^	69.0 ± 0.7
DSS50	49.0 ± 6.7 ^a^	26.8 ± 4.1 ^a^	60.0 ± 3.8	47.5 ± 3.0	41.8 ± 2.6	87.3 ± 0.9 ^ab^	65.8 ± 5.5
DS25	42.0 ± 4.8 ^ab^	23.0 ± 4.8 ^ab^	58.5 ± 4.8	49.5 ± 3.7	41.3 ± 3.5	86.3 ± 0.9 ^ab^	70.3 ± 1.3
DS50	49.0 ± 2.4 ^a^	20.8 ± 3.4 ^ab^	55.3 ± 3.4	42.8 ± 2.7	36.5 ± 2.3	83.5 ± 1.3 ^b^	65.8 ± 2.1

Percentage of spermatozoa (mean ± SEM). *n* = 4. The evaluation value of sperm motility parameters includes total motility (TM), progressive motility (PM), curvilinear velocity (VCL), velocity average path (VAP), velocity straight line (VSL), linearly (LIN), straightness (STR). ^a,b^ represents the significant difference (*p* < 0.05).

**Table 4:** Effect of different concentrations of djulis extract on viability and acrosome integrity of boar thawed semen.

Treatment	Viability (%)	Acrosome Integrity (%)
Control	57.0 ± 5.0	74.0 ± 6.0
DSS25	47.0 ± 3.0	70.0 ± 5.0
DSS50	46.0 ± 4.0	69.0 ± 12.0
DS25	51.0 ± 7.0	73.0 ± 7.0
DS50	46.0 ± 1.0	72.0 ± 6.0

Percentage of spermatozoa (mean ± SEM). *n* = 4.

## Data Availability

The original contributions presented in this study are included in the article; further inquiries can be directed to the corresponding authors.

## References

[1] Luño V., Gil L., Olaciregui M., Jerez R.A., de Blas I., Hozbor F. (2015). Antioxidant effect of lemon balm (*Melissa officinalis*) and mate tea (*Ilex paraguensys*) on quality, lipid peroxidation and DNA oxidation of cryopreserved boar epididymal spermatozoa. Andrologia.

[2] Gale I., Gil L., Malo C., González N., Martínez F. (2015). Effect of Camellia sinensis supplementation and increasing holding time on quality of cryopreserved boar semen. Andrologia.

[3] Brouwers J.F., Silva P.F.N., Gadella B.M. (2005). New assays for detection and localization of endogenous lipid peroxidation products in living boar sperm after BTS dilution or after freeze-thawing. Theriogenology.

[4] Lucio C.F., Regazzi F.M., Silva L.C.G., Angrimani D.S.R., Nichi M., Vannucchi C.I. (2016). Oxidative stress at different stages of two-step semen cryopreservation procedures in dogs. Theriogenology.

[5] Buhr M.M., Curtis E.F., Kakuda N.S. (1994). Composition and behavior of head membrane lipids of fresh and cryopreserved boar sperm. Cryobiology.

[6] Moran J.M., Madejón L., Ortega Ferrusola C., Peña F.J. (2008). Nitric oxide induces caspase activity in boar spermatozoa. Theriogenology.

[7] Santiso R., Tamayo M., Gosálvez J., Johnston S., Mariño A., Fernández C., Losada C., Fernández J.L. (2012). DNA fragmentation dynamics allows the assessment of cryptic sperm damage in human: Evaluation of exposure to ionizing radiation, hyperthermia, acidic pH and nitric oxide. Mutat. Res..

[8] Uribe P., Boguen R., Treulen F., Sánchez R., Villegas J.V. (2015). Peroxynitrite-mediated nitrosative stress decreases motility and mitochondrial membrane potential in human spermatozoa. Mol. Hum. Reprod..

[9] De Lamirande E., Jiang H., Zini A., Kodama H., Gagnon C. (1997). Reactive oxygen species and sperm physiology. Rev. Reprod..

[10] Aitken R.J., Ryan A.L., Baker M.A., McLaughlin E.A. (2004). Redox activity associated with the maturation and capacitation of mammalian spermatozoa. Free Radic. Biol. Med..

[11] Hussain T., Kandeel M., Metwally E., Murtaza G., Kalhoro D.H., Yin Y., Tan B., Chughtai M.I., Yaseen A., Afzal A. (2023). Unraveling the harmful effect of oxidative stress on male fertility: A mechanistic insight. Front. Endocrinol..

[12] Bansal A.K., Bilaspuri G.S. (2010). Impacts of oxidative stress and antioxidants on semen functions. Vet. Med. Int..

[13] Koziorowska-Gilun M., Koziorowski M., Fraser L., Strzezek J. (2011). Antioxidant defence system of boar cauda epididymidal spermatozoa and reproductive tract fluids. Reprod. Domest. Anim..

[14] Ros-Santaella J.L., Pintus E. (2021). Plant extracts as alternative additives for sperm preservation. Antioxidants.

[15] Tsai P.J., Sheu C.H., Wu P.H., Sun Y.F. (2010). Thermal and pH stability of betacyanin pigment of Djulis (*Chenopodium formosanum*) in Taiwan and their relation to antioxidant activity. J. Agric. Food Chem..

[16] Tsai P.J., Chen Y.S., Sheu C.H., Chen C.Y. (2011). Effect of nanogrinding on the pigment and bioactivity of Djulis (*Chenopodium formosanum* Koidz.). J. Agric. Food Chem..

[17] Chen S.Y., Chu C.C., Chyau C.C., Fu Z.H., Duh P.D. (2018). Effect of water extract of Djulis (*Chenopodium formosaneum*) and its bioactive compounds on alcohol-induced liver damage in rats. Int. J. Food Sci. Nutr..

[18] Chu C.C., Chen S.Y., Chyau C.C., Fu Z.H., Liu C.C., Duh P.D. (2016). Protective effect of Djulis (*Chenopodium formosanum*) and its bioactive compounds against carbon tetrachloride-induced liver injury, in vivo. J. Funct. Foods.

[19] Chyau C.C., Chu C.C., Chen S.Y., Duh P.D. (2015). Djulis (*Chenopodium formosaneum*) and its bioactive compounds protect against oxidative stress in human HepG2 cells. J. Funct. Foods.

[20] Chen S.Y., Chu C.C., Chyau C.C., Yang J.W., Duh P.D. (2019). Djulis (*Chenopodium formosanum*) and its bioactive compounds affect vasodilation, angiotensin converting enzyme activity, and hypertension. Food Biosci..

[21] Pursel V.G., Johnson L.A. (1975). Freezing of boar spermatozoa: Fertilizing capacity with concentrated semen and a new thawing procedure. J. Anim. Sci..

[22] Moraes C.R., Runcan E.E., Blawut B., Coutinho da Silva M.A. (2019). Technical Note: The use of iSperm technology for on-farm measurement of equine sperm motility and concentration. Transl. Anim. Sci..

[23] Matsuura K., Huang H.W., Chen M.C., Chen Y., Cheng C.M. (2017). Relationship between Porcine Sperm Motility and Sperm Enzymatic Activity using Paper-based Devices. Sci. Rep..

[24] Matsuura K., Wang W.H., Ching A., Chen Y., Cheng C.M. (2019). Paper-Based Resazurin Assay of Inhibitor-Treated Porcine Sperm. Micromachines.

[25] Carvajal G., Cuello C., Ruiz M., Vázquez J.M., Martínez E.A., Roca J. (2004). Effects of centrifugation before freezing on boar sperm cryosurvival. J. Androl..

[26] Sanocka D., Kurpisz M. (2004). Reactive oxygen species and sperm cells. Reprod. Biol. Endocrinol. RBE.

[27] Mokra D., Joskova M., Mokry J. (2022). Therapeutic effects of green tea polyphenol (−)-Epigallocatechin-3-Gallate (EGCG) in relation to molecular pathways controlling inflammation, oxidative stress, and apoptosis. Int. J. Mol. Sci..

[28] Ullah A., Munir S., Badshah S.L., Khan N., Ghani L., Poulson B.G., Emwas A.H., Jaremko M. (2020). Important flavonoids and their role as a therapeutic agent. Molecules.

[29] Han M., Zhang C., Suglo P., Sun S., Wang M., Su T. (2021). l-Aspartate: An essential metabolite for plant growth and stress acclimation. Molecules.

[30] Rahman S.U., Huang Y., Zhu L., Feng S., Khan I.M., Wu J., Li Y., Wang X. (2018). Therapeutic role of green tea polyphenols in improving fertility: A review. Nutrients.

[31] Wu M., Ni L., Lu H., Xu H., Zou S., Zou X. (2020). Terpenoids and their biological activities from Cinnamomum: A review. J. Chem..

[32] Pisoschi A.M., Pop A., Cimpeanu C., Predoi G. (2016). Antioxidant capacity determination in plants and plant-derived products: A review. Oxid. Med. Cell. Longev..

[33] Ye J.W., Ong W.A., Chao Y.Y. (2021). Analysis of antioxidant capacity of different colour strain of Djulis (*Chenopodium formosanum* Koidz.). Int. J. Agric. Innov. Technol. Glob..

[34] Li P.H., Chan Y.J., Lu W.C., Huang D.W., Chang T.C., Chang W.H., Nie X.B., Jiang C.X., Zhang X.L. (2020). Bioresource utilization of Djulis (*Chenopodium formosanum*) biomass as natural antioxidants. Sustainability.

[35] Yang S.M., Wang T., Wen D.G., Hou J.Q., Li H.B. (2016). Protective effect of Rhodiola rosea polysaccharides on cryopreserved boar sperm. Carbohydr. Polym..

[36] Chanapiwat P., Kaeoket K. (2015). The effect of Curcuma longa extracted (curcumin) on the quality of cryopreserved boar semen. Anim. Sci. J. = Nihon Chikusan Gakkaiho.

[37] Lee H.L., Lin M.Y., Wang H.S., Hsu C.B., Lin C.Y., Chang S.C., Shen P.C., Chang H.L. (2022). Direct-maternal genetic parameters for litter size and body weight of piglets of a new black breed for the Taiwan black hog market. Animals.

[38] Sharafi M., Borghei-Rad S.M., Hezavehei M., Shahverdi A., Benson J.D. (2022). Cryopreservation of semen in domestic animals: A review of current challenges, applications, and prospective strategies. Animals.

